# Gleanings in Science and Medicine

**Published:** 1893-09-09

**Authors:** 


### Gleanings in Science and Medicine.

A novel method of cleaning out wells impregnated with foul air was adopted not long since, and was described in the pages of a weekly contemporary. The man who had undertaken to execute this work commenced it by first extracting the poisonous gas at the bottom of the well through the medium of an ordinary umbrella, which he let down attached to a string. Carbonic acid gas being heavier than air, the man was able to fill and refill the umbrella, and thus clear the well of its more poisonous contents ere he himself descended to complete the work.

The Local Government Board has issued a "further report and papers" on the influenza epidemic from 1889-1892, which period they have sub-divided into, firstly, the winter of 1889-90; secondly, April to June, 1891; and, thirdly, the winter of 1891-92. The comparative fatality of the three periods differs very considerably according to locality. In London the death rates from influenza in the three years of 1890, 1891, and 1892 were 146, 544, and 531 per million living respectively. The onset of the disease was most sudden in 1889, but while there were a greater number of persons attacked, the fatalities were fewer. In other places, however, precisely the reverse was noticed. In Sheffield, for example, the first attack was mild and protracted, while in 1891 it was sudden, severe, and fatal. No conditions of site, soil, climate, sanitary circumstances, or occupation could give any clue to the reason of these differing incidences; but there is a certain amount of evidence proving that the influence of a severe epidemic serves to grant some sort of immunity against another in the same place.

The Pharmaceutical Society of Ireland has done a good work lately by exposing and holding up to public scorn, the somewhat audacious quackery of a certain well-advertised complexion doctor, of Bond Street fame. Madame Anna Rupert stands convicted at Dublin with selling a skin specific containing poison (about eight grains of corrosive sublimate in solution) for which, as for other commodities, she had no licence under the Pharmacy Act. The particular preparation brought before the Court was a recognised remedy for removing freckles, but one requiring to be used with caution, and under medical direction; indeed, in Taylor's work on "Medical Jurisprudence," two cases are quoted wherein death was caused by doses containing three grains less than those in "Rupert's Skin Tonic"—a tonic by the way which was declared to be "harmless" on the self-constituted authority of the originator. The fact that the ingredients of the bottle which was thus advertised at 10s. 6d. were worth about 4d. is of small consequence. If the public will submit to such extortion it is its own affair; but the voices of all right-thinking men cannot be too strongly raised against the roguery which sells, and the folly which buys, remedies for those evils which should only be treated under skilled professional advice.

Berlin hospitals are not yet to open their wards to clinical teaching; a fresh attempt to induce the authorities to extend the sphere of hospital usefulness in this direction has again been met by a refusal. The corporation of Berlin appear to be obdurate against this much-needed reform in matters medical; for to all others but this august body themselves it appears to be a wasted opportunity that the buildings which the University have devoted to surgery and to the diseases of women should not be put to a twofold advantage, and combine also the possibility of a clinical education for students. The "Charité," meanwhile, is becoming overloaded, and can ill meet the requirements of students, who in such numbers now crowd its insufficient accommodation.

An Italian doctor of much repute has lately published a report on certain investigations which he undertook, relating to the effects of tobacco smoke on microbes. The general summary of his researches (without going into detail) goes to prove that the smoke of the Cavour, Virginia, and Tuscan cigars, as all black and chopped tobaccos, possesses a very pronounced bactericide power, acting particularly on the bacillus of Asiatic cholera. In epidemics of typhoid fever, as also of cholera, the doctor asserts the use of tobacco may be useful rather than harmful; whilst in the hygiene of the mouth tobacco smoke merits special consideration, as a prophylactic means of combating microbian affections of the buccal cavity. Scientists for the most part will be in sympathy with the views which Dr. Tassionari has thus formed, after a long and laborious study, of the bactericidal value of tobacco; yet we cannot doubt that many rabid non-smokers will be found ready to combat the fact that any effective good can come from indulgence in so pernicious a habit.

In view of the many schemes for temperance legislation which throng the political horizon, the report of the Collective Investigation Committee of the British Medical Association on the subject of "Temperance and Health" possesses a special interest for us. The committee who undertook this work divided the subjects of their investigation into these classes, *i.e.*, total abstainers, habitually temperate, careless drinkers, and the decidedly intemperate. Resultant on their labours in this field of research the committee give the following table, illustrative of the relative longevity of the persons thus classified:—

| | |
|---|---|
| Habitually temperate ... ... ... | 62·13 years. |
| Careless drinkers ... ... ... | 59·67 ,, |
| Free drinkers ... ... ... ... | 57·59 ,, |
| Decidedly intemperate... ... ... | 52·03 ,, |
| Total abstainers... ... ... ... | 51·22 ,, |

Here we see that the respective chances of long life are more in favour of the hopeless drunkard than of the actual teetotaler, and the advocates of temperance will find it hard to deny the accuracy of these figures, which are published under the ægis of a qualified body of medical experts.

